# “We are all one together”: peer educators’ views about falls prevention education for community-dwelling older adults - a qualitative study

**DOI:** 10.1186/s12877-015-0030-3

**Published:** 2015-03-20

**Authors:** Linda Khong, Fiona Farringdon, Keith D Hill, Anne-Marie Hill

**Affiliations:** Institute for Health Research, School of Physiotherapy, The University of Notre Dame Australia, PO Box 1225, Fremantle, Western Australia 6959 Australia; Institute for Health Research, School of Health Sciences, The University of Notre Dame Australia, PO Box 1225, Fremantle, Western Australia 6959 Australia; School of Physiotherapy and Exercise Science, Curtin University, GPO Box U1987, Perth, Western Australia 6845 Australia

**Keywords:** Aged, Aged 80 and over, Health promotion, Accidental falls, Interview, Focus group, Peer group, Volunteer workers, Health education, Qualitative research

## Abstract

**Background:**

Falls are common in older people. Despite strong evidence for effective falls prevention strategies, there appears to be limited translation of these strategies from research to clinical practice. Use of peers in delivering falls prevention education messages has been proposed to improve uptake of falls prevention strategies and facilitate translation to practice. Volunteer peer educators often deliver educational presentations on falls prevention to community-dwelling older adults. However, research evaluating the effectiveness of peer-led education approaches in falls prevention has been limited and no known study has evaluated such a program from the perspective of peer educators involved in delivering the message. The purpose of this study was to explore peer educators’ perspective about their role in delivering peer-led falls prevention education for community-dwelling older adults.

**Methods:**

A two-stage qualitative inductive constant comparative design was used. In stage one (core component) focus group interviews involving a total of eleven participants were conducted. During stage two (supplementary component) semi-structured interviews with two participants were conducted. Data were analysed thematically by two researchers independently. Key themes were identified and findings were displayed in a conceptual framework.

**Results:**

Peer educators were motivated to deliver educational presentations and importantly, to reach an optimal peer connection with their audience. Key themes identified included both personal and organisational factors that impact on educators’ capacity to facilitate their peers’ engagement with the message. Personal factors that facilitated message delivery and engagement included peer-to-peer connection and perceived credibility, while barriers included a reluctance to accept the message that they were at risk of falling by some members in the audience. Organisational factors, including ongoing training for peer educators and formative feedback following presentations, were perceived as essential because they affect successful message delivery.

**Conclusions:**

Peer educators have the potential to effectively deliver falls prevention education to older adults and influence acceptance of the message as they possess the peer-to-peer connection that facilitates optimal engagement. There is a need to consider incorporating learnings from this research into a formal large scale evaluation of the effectiveness of the peer education approach in reducing falls in older adults.

## Background

Falls in older adults are a significant health problem, with approximately one third of community-dwelling older adults falling once or more annually [1,2]. Falls are the most frequent cause of injury related hospitalisation for older people in Australia with one in every 10 days spent in hospital by a person aged 65 and older in 2009–10 directly attributable to an injurious fall [3]. Recent meta-analyses concluded that there are effective interventions such as exercise that reduce both the rate and risk of falls in community-dwelling older adults [4]. Despite this, the lower than optimal level of uptake and adherence to falls prevention strategies in clinical trials [5] demonstrated a gap in translation of this evidence into practice among the older adult population. Barriers to the engagement and uptake of falls prevention strategies may include the perceptions and beliefs of older adults. Studies have found that older adults have low awareness of effective falls prevention strategies, nominate barriers to engagement in such strategies and may perceive these strategies to be relevant to other older people, not themselves [6-10].

Peer-led education is one recommended approach to facilitate translation of falls prevention messages to community-dwelling older adults [11,12]. Peer education has been described as an “umbrella term” involving a range of interventions where information, values or behaviours are conveyed between the educator and peers who share similarities such as age or shared experiences [13,14]. A systematic review found that peer education can be effective in promoting health behaviour change such as self-efficacy in chronic disease and self-management [15] but studies reviewed included younger participants and also one-on-one peer education formats that may sometimes be delivered electronically. The effectiveness of peer education has not been well investigated in the area of older adults and falls prevention, in particular peer education delivered in a one-to-group format. Peer educators are deemed to be positive role models who could influence and improve behavioural outcomes, encourage self-efficacy and foster a sense of empowerment about falls prevention amongst their peers [11,16]. Behaviour change models also suggest that factors such as being a credible source, role modelling and instructions to perform the new behaviour may be persuasive in promoting positive behavioural outcomes [17,18]. Peer educators demonstrate positive social identity related to falls prevention which is important because previous studies have found that older adults perceive falls inevitable and suggest that there is nothing that can be done to prevent falls [6,10,19] by older adults. The peer educators may facilitate empowerment within their audience and may encourage older adults to view falls as preventable. Furthermore, for adults to adopt behaviour change, the health information should be delivered in a manner consistent with adult learning principles [20,21]. These principles include recognising that adults are self-directed learners who need to understand the rationale for what they are learning.

Few studies have investigated the impact of peer education on falls outcomes. In one study, 26% of older adults who received falls prevention peer education made changes to reduce their risk of falling [22]. In other studies, peer education resulted in uptake of falls prevention actions but there was no significant reduction in falls [23,24]. However, these studies did not explore the views of peer educators about their role in delivering falls prevention messages to an older adult audience and their perceptions about how falls prevention messages can be delivered effectively to promote behaviour change. Previous studies have also not specifically investigated the role and relationship of peer educators and of their coordinating organisation.

Therefore, the purpose of the study was to explore the perspectives of a group of peer educators about their role in delivering peer-led falls prevention education for community-dwelling older adults and subsequently, to inform future refinements for peer education falls prevention programs.

## Method

### Study design

**Figure 1:**
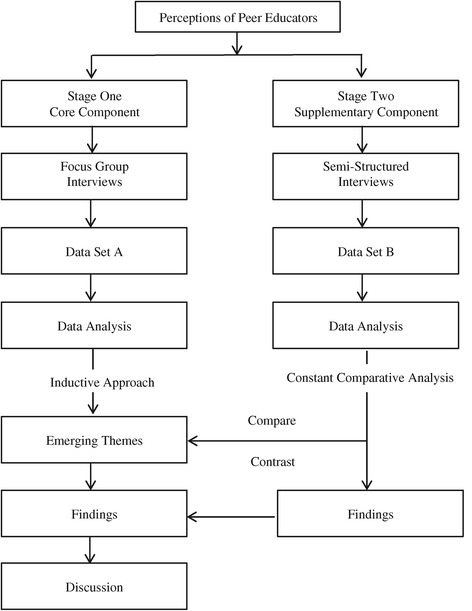
**Research design for exploring the perceptions of peer educators about delivering falls prevention education to community-dwelling older adults.**

A two-stage qualitative inductive constant comparative design [25,26] was used (Figure 1). This design was chosen to gain an in-depth understanding of the numerous interpretations from the peer educators about the program in which they were involved. In Stage One, focus group interviews (core component) were used to gain the peer educators’ perspectives of their role and effectiveness in delivering the falls prevention message. The emerging categories identified from the preliminary analysis of data obtained from these focus group interviews were further explored in subsequent Stage Two semi-structured interviews (supplementary component) to elicit a broader and more in-depth scope to the preliminary findings [27].

### Sample

A purposeful sample was recruited consisting of all peer educators who delivered falls prevention presentations to groups of community-dwelling older adults living in Perth metropolitan areas, Western Australia (WA). In this study, peer education was defined as a one-peer-to-one group approach, delivering a one-off session on falls prevention health-related education.

Peer educators in the program are volunteers who are trained to conduct presentations and raise awareness of falls prevention to the broader community. They are mostly retired and highly educated older adults who choose to volunteer to contribute to the community. They form part of a large community organisation that focuses on providing education, promotion and resources directed towards injury prevention and community safety in WA. The community organisation recruits interested older adults who are keen to volunteer for this peer education program as well as their other volunteer activities. Falls prevention is a key focus of the organisation and it conducts training for new peer educators to deliver falls prevention education presentations. The organisation also organises the schedule of presentations and provides support and resources for the peer educators.

### Ethical considerations

Ethics approval was obtained from The University of Notre Dame Australia Human Research Ethics Committee (Reference Number: 013061 F) and all participants gave their written consent prior to data collection.

### Data collection and procedure

Data collection occurred in two stages. In Stage One, the focus group interview technique was used as a method of collecting multiple perspectives in a single short session [28].

All peer educators (n = 11) were invited by the community partner’s program training coordinator to participate in focus group interviews and all accepted. The focus group participants’ profile and characteristics are presented in Table 1. Eight (73%) of the participants were less than 75 years old and five (46%) had delivered peer education presentations for more than five years. Four (36%) identified themselves having a background in education.

**Table 1:** **Demographic profile of the focus group participants**

	**n = 11 (%)**
**Age**	
65-74 years old	8 (73)
75-84 years old	2 (18)
85+ years old	1 (9)
**Gender**	
Female	7 (64)
Male	4 (36)
**Education**	
Secondary	5 (46)
Trade	1 (9)
Diploma	1 (9)
University	4 (36)
**Status**	
Completely retired	9 (82)
Partly retired	2 (18)
**Former Primary Employment**	
Education	4 (36)
Health	2 (18)
Business and Legal	4 (36)
Manufacturing	1 (9)
**Years as Volunteer Peer Educator**	
11 months of less	1 (9)
1-5 years	5 (46)
6+ years	5 (46)
Note: Data Analysis by SPSS Version 22	

Two focus groups were conducted in May 2013. Group one was comprised of six participants and group two was comprised of five participants. Focus group interviews were conducted at the community organisation’s office meeting room where the peer educators meet on a regular basis, thereby ensuring the study’s participants would find the surroundings familiar to them and be comfortable. The interviews were also held at dates convenient for the peer educators to maximise participation. The two researchers who conducted the focus groups were physiotherapists who were experienced in management of falls prevention programs for older people. Both researchers also had training in qualitative methods of research including conducting focus group and interviewing methods. At the commencement of each focus group interview, the focus group facilitator (primary researcher) introduced herself and her fellow researcher co-facilitator and note taker, explained the aim of the interview and the process of audio-taping and ongoing note-taking and sought an undertaking of confidentiality. Each focus group interview lasted about one hour in duration. Refreshments were provided throughout the discussions. The note taker discussed immediate, significant moments with the primary researcher while on-site after completion of each focus group interview.

The researcher used an open-ended interview guide that was informed by research in peer education with older adults in the area of health [29-31] and by an expert in falls prevention. The guide was further reviewed by the program training coordinator, thereby ensuring the trustworthiness of the data within the context of delivering peer-led falls prevention education. The interview schedule included open-ended questions related to the following:

- Role of peer educator
- Skills that they perceived as being required in their role as peer educator
- Challenges they faced as a peer educator and strategies they employed to address those challenges
- Factors they felt influenced their effectiveness in delivery of their presentation
- Feedback about how they perceive the support from the community organisation in provision of training and resources in their role as peer educator.

Stage Two’s supplementary component included two participants who were invited to participate in a one-on-one semi-structured interview to confirm and further explore preliminary findings from the focus groups. Their selection was based on their insights they provided during the focus group interviews.

Each semi-structured interview was an hour in duration and was undertaken in a meeting room at the community organisation. The semi-structured interview involved a combination of conversational strategy within an interview question guide approach [32]. The questions were identified from the focus group findings. They were open-ended and phrased to explore the topics in further scope and depth with the participant. The interviewer, a physiotherapist experienced in falls prevention with older people, summarised the main points at the end of each interview to seek final verification from the participant.

### Data analysis

Data sets and analyses for the core and supplementary components were kept separate until the analyses were completed then each analysis was incorporated into the results narrative (Figure 1). The data from the supplementary component verified and added scope to the findings of the core component.

### Focus group

**Figure 2:**
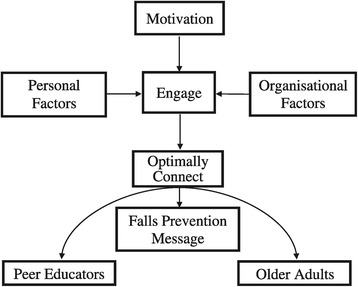
**Conceptual framework describing peer educators’ perceptions of the mechanism of how they deliver the falls prevention message to older adults**.

The primary researcher first listened to the focus group interview audiotapes before transcribing them verbatim. Pseudonyms were assigned to participants for confidentiality. The data were managed using the software tool NVivo10 for Windows [33]. An inductive approach using thematic analysis of the content of the focus group transcripts was applied to analyse the data [34,35]. Two researchers conducted data reduction and data analysis independently and then met to finalise codes and themes through discussion to arrive to a consensus. This provided investigator triangulation [36], which aimed to increase the rigour of the analyses. Preliminary emerging themes (patterns) were visually displayed in a conceptual framework [35] (Figure 2). Subsequently the primary researcher met with the focus group participants to seek verification of the emerging themes. Any differences between the participants and the researchers were resolved by consensus.

### Interviews and integration with focus groups

The data from Stage Two’s semi-structured interviews were then analysed by the two researchers who independently coded the data and compared emerging themes with those that emerged from the focus group interviews. This methodological triangulation was intended to increase the rigour of the findings. The conceptual framework (Figure 2) was developed from the focus group interviews and confirmed by the semi-structured interviews.

## Results

Thematic analyses identified that peer educators were motivated to present their message and sought to engage in a manner that optimally connected them with their older adult audience. They perceived that this was essential to facilitate acceptance of the falls prevention message. Two main categories were identified as affecting the levels of engagement with the older adults that was achieved; these were personal factors and organisational factors (Figure 2).

### Motivation

The focus group participants reported that they were enthusiastic to share the falls prevention message with their peers because they perceived through their own experience, including through family and friends that the message was personally relevant “this message has done me a lot of good” (Participant 10) and “I would like to see it succeed as so many people I know have had falls” (Participant 1). They also described strong personal motivation and enthusiasm for volunteering to deliver falls prevention messages as “I considered it was up to me to give something back” (Participant 11). Supplementary interviews with Participant 1 and 5 confirmed the focus group findings regarding motivation to deliver the falls prevention message. They expressed strong beliefs that the falls prevention message was important and was a worthwhile program to be disseminating in the community. One participant shared:

*“I would like to see it succeed because most of the people I know have had falls including myself and some of the factors that come into the talks are so simple to implement it is such a shame if we don’t get that message out*.” (Participant 1)

### Personal factors

Personal factors that facilitated message delivery and engagement included peer-to-peer connection and perceived credibility, while barriers included limited access to resources.

#### To engage using the peer-to-peer connection

The focus group participants perceived that as peers they were able to engage in a peer-to-peer communication with their older adult audience because they could strongly relate to them “we are all one together” (Participant 5)*.* They shared that they felt able and comfortable to encourage their older adult audience with “we can do this” instead of “you can do this” (Participant 5) because of the peer-to-peer connection and as a role model because “I can do it, you can do it” (Participant 11). They described peer-related humour and anecdotal examples during presentations that they used to capture their audience’s attention.

Participants 1 and 5 (supplementary interviews) elaborated that they were in a better position to communicate and deliver the message than a younger person because they could connect to their audience as peers “it is that relating, that we are all doing the same sort of things or at the same stage of life” (Participant 5). In addition, they felt that it would be ideal to communicate and emphasise the health, social and emotional consequences of a fall at this older stage of life with “a disruption to your life, and your family’s lives, the costs associated with it as well as time and intrusions” but “without actually frightening people” (Participant 1). These two participants also expressed a desire to further engage their older adult audience in their message by goal-setting and action planning with “those who came can take home three points that they then apply to their life and so get benefit from” (Participant 5).

#### To engage with credibility

Credibility was identified as being important in delivering the falls prevention message and there was a strong recommendation that preparation and planning for each presentation was required. The focus group participants advised “make sure you are familiar with the material and able to answer questions” (Participant 4). They also described being proactive in acquiring hands-on presentation skills through observing more experienced peer educators. These focus group participants emphasised repeatedly that they required access to current evidence and information for their presentations, as it “can be embarrassing if you are challenged and your data is wrong” (Participant 1). However establishing credibility was also perceived as “being as good an example as I can be” (Participant 1) and “the need to be role models in terms of how we go about things” (Participant 4).

#### Perceived barriers to engagement

The focus group participants nominated perceived barriers in engaging the audience in the presentations, which they reflected could possibly contribute to the audience’s willingness to take on the falls prevention message. They explained that these barriers included the perceived receptiveness from an older adult audience, time limitations for the presentations and access to equipment at the venues.

Anecdotal feedback they received regarding some presentations indicated that the older audience did not think that falls would happen to them in that “they say we’re too active that’s not going to happen to us so you can get that resistance” (Participant 6). Similar to the focus groups, the interviewed participants reiterated that an older people’s approach is “it won’t happen to me” (Participant 5) or “that was interesting… I don’t think they do anything about it” (Participant 5). They were keen to “to make them aware that they are at risk” (Participant 5). One interviewed participant elaborated on the rationale of the peer educators’ desire to address a “younger audience” with comments such as “we are chipping around the edges.....we have to get to the younger audience to actually prevent people that start to fall, at 60 or 60ish” (Participant 1).

However this barrier did not lower the peer educators’ motivation because “even if you reach one person and stop one person from falling, that’s something” (Participant 11).

As older adults, the peer educators also recognised that some of the audience may have age related changes which could affect their ability to understand the presentation and hence receive the falls prevention messages “we must realize the difference in age as to retention levels and ability to perform” (Participant 9). Some participants expressed a degree of uncertainty about the effect of their falls prevention presentation with comments such as “when you give information handouts whether they take them home and read them”. (Participant 3). The limited time available to present when considering the scope of the falls prevention message was viewed as affecting how well they could engage with their audience as “to get through all of this in 30 minutes’ presentation, this is impossible” (Participant 9).

*“If it’s at a senior centre, the’ve got half an hour time between this activity and lunch, all you’re doing is just presenting, you’re not engaging that audience, you are not getting a transfer of learning taking place.”* (Participant 9)

Finally, the falls prevention presentations were delivered in a broad range of settings in the community and this meant that “not every group has the equipment that you are able to use or equipment may not be in working condition” (Participant 7). It was suggested that this barrier could be overcome with flexibility and willingness to adjust “you need to be able to take over and present different parts of the presentation if the equipment does not work when you get there” (Participant 1).

### Organisational factors

The organisational factors category reflected peer educators’ perceptions about how the community organisation provided support them to deliver an effective falls prevention message. These emerging themes were ongoing training and formative feedback following the peer educators’ presentations, resources for the audience and audience profile.

#### To support with training and feedback

The participants’ views about training were influenced by their life skills, work and personal experience (Table 1) before retirement. Presentations were viewed as a “combination of information and delivery” (Participant 2) and it was perceived that each needed to be optimal if they were to effectively deliver their falls prevention message. Therefore, the focus group participants expressed strong interest in receiving formative feedback about their delivery. However, there was considerable debate amongst these participants whether formative feedback should be from the community organisation or from an external party deemed more suitably qualified to assess their delivery. They further elaborated that feedback on their presentations needed to be constructive in terms of “direction on how you might improve if you need to” or “this area needs to be, like toughened up, changed or altered” (Participant 2). The peer educators were also keen to obtain meaningful feedback from the audience so that they could feel more assured and improve in “getting the message over” (Participant 11).

In addition, the interviewed participants further elaborated and were also consistent in their suggestions about requiring support and expressing a desire to undertake further training for their role.

*“I would like somebody who is a public speaker to come out and sit in on a talk, maybe once a year and give some feedback as to how it is going and how it could be improved so that would be support”* (Participant 1)*.*

Other suggested training opportunities included “fine-tune it at volunteer meetings” (Participant 5) and seeking further feedback to upgrade their skills. They thought it could include the audience’s feedback from their presentations. Specifically, “if it was something I wasn’t doing or wasn’t getting the message across and of course, the positive things like they learned a lot” (Participant 5).

Furthermore, the two interviewed participants also suggested that new peer educators could benefit from more training including “a bit more theory” as “the more knowledge they have, the better they can present it” (Participant 5). They considered that having more structure is helpful for those who have not had much prior knowledge or presentations. Flexibility in delivery was identified as being beneficial for those with knowledge and confidence.

#### To support with resources

The majority of focus group participants strongly emphasised that the resources (brochures, videos, questionnaires) should be “up-to-date” and at an appropriate level of comprehension for the older adult audience. Again as older adults they recognised that “we cannot make an assumption that they can all read and write and comprehend exactly as we do” (Participant 2).

A minority of participants identified that the falls message could require tailoring to enhance learning “we’ve got to sum up that group, we can’t deliver the same message, the same way to different groups of people, it is impossible” (Participant 9). These participants suggested that catering to audience’s different learning styles could promote the learning experience including the use of resources and equipment as “supporting material in trying to use different senses” (Participant 9) and “there’re some people that listen but there are some people that are visual and for some people being able to see it makes the impact” (Participant 3).

#### To support with appropriate audience

There was strong feedback from focus group participants that the falls prevention presentations should “target the audience which is most likely to get a benefit from what we have to say” (Participant 2). They expressed the idea that a “slightly younger audience” (Participant 1) would also benefit more from the message and were “disappointed if they’re in their 70s, 80s and 90s which they often are” (Participant 1). Functional ability profile of the audience was another aspect considered important to maximise targeting of the falls prevention messages to an appropriate group of older people. Focus group participants recognised that their presentations were not targeted to an audience who were highly dependent in their mobility (such as those who are wheelchair bound or may come from a residential care setting).

## Discussion

The peer educators provided important insights regarding what their role entailed. They revealed the spectrum of practical and emotive dimensions they perceived influenced their capacity to effectively deliver falls prevention education to their older adult audience (peers). The conceptual framework suggests the educators perceived that a key aspect of their role was to engage and connect optimally on a personal level with their peers. This has been described as *peer connection* in a peer explanatory model [37] which conceptualises that it is this connection that creates a comfortable space for sharing and learning. The peer educator intuitively recognised the peer connection to engage their peers with the falls prevention message was needed as a precursor because provision of information alone was not going to be effective in achieving behaviour change. The educators expressed the belief that the peer-to-peer communication and engagement with their peers could improve the level of acceptance and future uptake of falls prevention message and strategies. This belief is supported by health behaviour concepts [38] and adult learning theory [21]. Although capability and knowledge are required to change health behaviours, the engagement and motivation of the target audience is an essential component which facilitates uptake of health behaviour [38].

These peer educators reflected on their own experience as an older adult in recognising that their peer audience may have had low self-awareness about falls and low levels of motivation to engage with messages about falls prevention. This rationale is consistent with previous findings that older people often do not see the personal relevance of falls prevention messages [10]. The peer educators identified that their peers in the audience were likely to have the view that falls happen to others and not themselves. This was also identified in large studies that have explored older adults’ self-perceived risk of falls [9,39,40]. The peer educators perceived this as one of the barriers to acceptance of their falls prevention message, and this could be the reason they sought more support in addressing these barriers.

Consistent with behaviour change framework and techniques [18,38,41], the peer educators identified strategies to overcome these potential barriers. In addition to provision of information on how to minimise the risk of falls, concepts of persuasion, credibility and modelling in their delivery of the message were identified as ways to influence their peers’ perceptions. Furthermore, the peer educators reported managing any prevailing perceived low self-efficacy [39] amongst their peers by role modelling*,* proposed by the Social Cognition Theory [17]. This strategy and others aimed to persuade and to empower their peers’ self-belief in their own capacity to succeed in taking steps to reduce their risk of falling. It was also deemed important by the peer educators that they were seen as a credible source to engage their peer which is a concept that is also supported by behaviour change theory [41]. To further support this credibility, organisational support was viewed as required to provide resources such as up to date falls prevention data.

Research in education [42,43] demonstrates that the educator’s level of motivation and enthusiastic behaviour may engage and influence the audience positively. Moreover, the peer educators portrayed themselves as adult learners and at the same time exhibited implicit awareness of key adult learning principles [20,21] in their work. They were self-motivated and self-directed in their learning such as learning from fellow peer educators in buddy-training and seeking formative feedback on their performance. Their insights about how to stay flexible, to tailor the message and seek access to resources in various sensory formats such as video and flyers to meet the audience’s different learning needs are consistent with adult learning theory [21,44]. These principles have been found to help improve the learning experience and subsequently, improve retention of information [45,46]. This can subsequently improve transfer in learning and acquiring knowledge and information [47], thus enhancing the opportunity towards achieving behaviour change [41].

The peer educators perceived that the community organisation with its focus on injury prevention was important in providing a mechanism to assess, train and provide timely formative feedback to enhance the performance of peer educators to optimally deliver the falls prevention message. However, they suggested that there could be value in providing additional tools and processes to empower the peer educators to cultivate their capabilities, and acquire new skills, which would sustain them to continue to deliver the falls prevention message to their peers

### Limitations of the study and future research directions

The researcher was seen as an ‘outsider’ to the peer educators and the community organisation and hence, the peer educators appeared to share and discuss their perspectives comfortably. However, the researcher developed a relationship with these peer educators over the course of this study, therefore some researcher bias may have influenced the analysis. However, the research included a second independent researcher coding the data (investigator triangulation) and a supplementary component (methodological triangulation) to minimise researcher bias in this qualitative study.

The purposive sampling was intended to seek an understanding of peer education in falls prevention by exploring these peer educators’ experience. The limited size of the participant numbers with only two focus groups meant that data saturation or redundancy may not have been reached. Although small, the sample included all peer educators associated with the peer educator program, so was considered comprehensive. These findings relate specifically to one peer education falls prevention program, and may not be generalisable to other falls prevention peer education programs elsewhere in Australia or overseas. Although the findings of this study have drawn attention to issues that are specific to this context of falls prevention education in the community, these findings are congruent with other current research in the area of education, falls prevention and adult learning. This study did not consider the perspectives of the community organisation’s staff involved with the peer education falls prevention program, nor those of the audience that attended the presentations. Further research investigating the target audience’s perspectives and a formal evaluation of the effectiveness of this type of peer education program to achieve behaviour change and reduce falls is required.

## Conclusion

Older adults who undertake the role of peer educators understand through their own experience that there are facilitators and barriers that can influence falls prevention messages being accepted by their peers. By engaging and optimally connecting with their peers, these peer educators aim to influence acceptance of the falls prevention message and subsequent behaviour change. Training, adoption of adult learning principles and timely feedback can affect optimal delivery of the falls prevention presentations.
